# Supplementary material for: Inflammatory factors and risk of lung adenocarcinoma: a Mendelian randomization study mediated by blood metabolites
Source: Front Endocrinol (Lausanne). 2024 Aug 27;15:1446863. doi: 10.3389/fendo.2024.1446863 (PMC11384989; doi:10.3389/fendo.2024.1446863)
Supplement: Supplementary file 1 [file DataSheet1.zip › supplementary files/S2/GCST90200434/sensitivity-analysis.pdf]

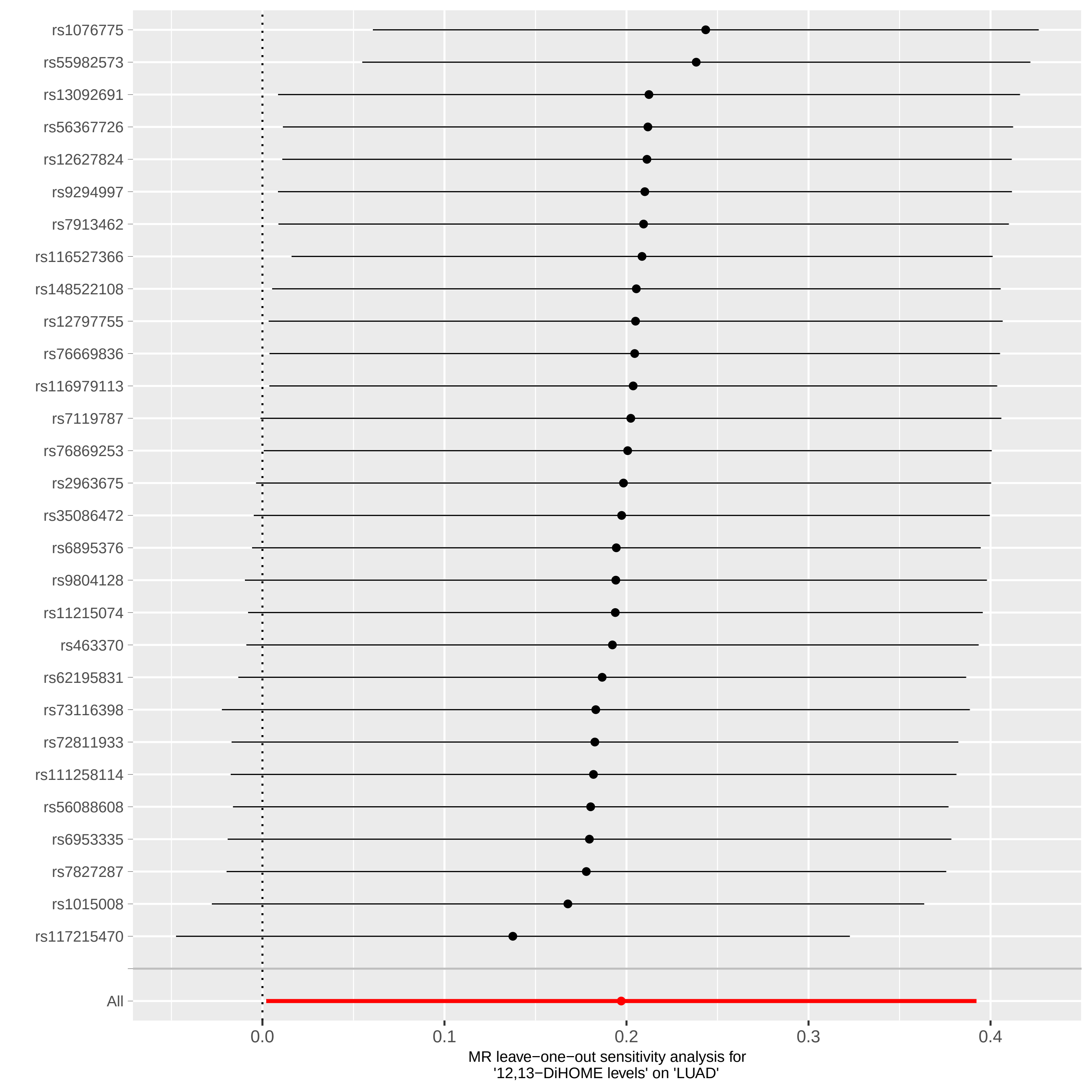

rs1076775
rs55982573
rs13092691
rs56367726
rs12627824
rs9294997
rs7913462
rs116527366
rs148522108
rs12797755
rs76669836
rs116979113
rs7119787
rs76869253
rs2963675
rs35086472
rs6895376
rs9804128
rs11215074
rs463370
rs62195831
rs73116398
rs72811933
rs111258114
rs56088608
rs6953335
rs7827287
rs1015008
rs117215470
All
0.0
0.1
0.2
0.3
0.4
MR leave−one−out sensitivity analysis for
'12,13−DiHOME levels' on 'LUAD'
